# Influence of Changes in the Level of Volatile Compounds Emitted during Rapeseed Quality Degradation on the Reaction of MOS Type Sensor-Array

**DOI:** 10.3390/s20113135

**Published:** 2020-06-01

**Authors:** Robert Rusinek, Henryk Jeleń, Urszula Malaga-Toboła, Marek Molenda, Marek Gancarz

**Affiliations:** 1 Institute of Agrophysics Polish Academy of Sciences, Doświadczalna 4, 20-290 Lublin, Poland; m.molenda@ipan.lublin.pl; 2 Faculty of Food Science and Nutrition, Poznań University of Life Sciences, Wojska Polskiego 31, 60-624 Poznań, Poland; henrykj@up.poznan.pl; 3 Faculty of Production and Power Engineering, University of Agriculture in Kraków, Balicka 116B, 30-149 Kraków, Poland; umalagatobola@gmail.com

**Keywords:** electronic nose, rapeseed, gas chromatography, ergosterol, smellprint

## Abstract

This study presents the applicability of a three-parameters method for digital description of spoiled rapeseed odor based on the use of an electronic nose. The method consists of the use of three parameters to describe the sensor response, i.e., the maximum resistance value, the response time and the cleaning time of the active surface of the sensor. Reference chemical methods, i.e., determination of the ergosterol content and analysis of volatile compounds by gas chromatography-mass spectrometry, were used to monitor qualitative changes occurring in the stored material. A 31-day profile of volatile compounds and changes in the ergosterol content was determined in the study. A total of 18 chemical groups of volatile organic compounds was identified. There was a strong positive correlation between the cleaning time and the percentage content of alcohols and alkenes, as well as ergosterol, as a marker of qualitative changes. The maximum response was another parameter that effectively described the changes occurring in the seeds. This parameter was strongly negatively correlated with esters and amides in the case of six sensors, and with ergosterol, alkenes and to a lesser degree with alcohols in the case of the other two sensors. The study results clearly demonstrated a relationship between the sensor responses and the percentage content of alcohols and alkenes, which provided novel practical information for the oilseed branch.

## 1. Introduction

Classical methods for analysis of gas substances are mainly based on the use of gas chromatography combined with mass spectrometry (GC-MS), ion mobility spectrometry (IMS), chemiluminescence techniques (CL), UV detection, Raman spectroscopy and olfactometric techniques. Despite the unquestionable advantages of the techniques used so far, new construction and apparatus solutions are sought to overcome some of the disadvantages associated with the use of classical methods of odor analysis. Devices that may complement them in the near future include chemical sensors or instruments consisting of a sensor matrix. A device that mimics the sense of smell in a simplified way is the electronic nose, which is increasingly being used. The electronic nose has been used for decades as an effective tool for non-invasive diagnostics in many areas of science and economy [1,2,3,4]. The first simple devices were constructed in the fifth decade of the last century. These devices have been applied as simple odor analyzers in medicine, food assessment, environmental tests, etc. [5,6,7,8,9]. The technological progress observed in recent decades has contributed to the development of hardware and software for e-nose techniques [10]. Generally, the attention of scientists and researchers is focused on the use of more advanced techniques for the analysis of large datasets, which will contribute to a broader application of this type of device in practice [11,12]. Additionally, it is possible to improve the functions of electronic noses and acquire larger amounts of data describing an odor during a single measurement of volatile organic compounds [13]. Therefore, every step leading to improvement of the devices and measuring methods seems to be important. Hence, to satisfy the demand for the development of techniques for volatile organic compounds (VOC) measurements with the use of electronic noses, we have devised a three-parameter method for the generation of smellprints useful in the identification of odors, and compared it with other commonly used methods [14]. This is a particularly important approach, since generally only one parameter, i.e., the maximum sensor response, is commonly used to describe odors. For precise validation of the common approach to electronic noses, which is to combine multiple features of the sensor response, we designed a comprehensive research program to correlate the classical methods of assessment of the quality of biomaterials with the three-parameter e-nose technique for odor description [15,16]. Following our previous experience, we chose rapeseed as a research material and subjected it to a controlled process of qualitative degradation, in order to obtain a variable odor profile correlated with the degradation level [17,18]. 

Rapeseed (*Brassica napus* L.) is one of the most popular oilseed plants in the European Union, especially in Poland, Germany, Great Britain and France. Rapeseed crops account for 10% of global oilseed production. In the era of the search for alternative energy sources, rapeseed oil is not only edible, but is also used for biodiesel production [19,20]. During the investigations, volatile compounds forming in the process of seed spoilage were identified using GC-MS, and changes in the content of ergosterol, i.e., an indicator of the presence of fungal biomass in stored materials, were determined. The goal of the experiments was to correlate specific chemosensitive sensor response parameters with changes in stored rapeseed measured with chemical methods, which are the “classic” methods in this type of research.

## 2. Materials and Methods

### 2.1. Preparation of Research Material

Spring oilseed rape cv. Sensatione was used in the experiments. The rapeseed was harvested in Poland and provided by the Institute of Agrophysics PAS, Poland, in 2019. To obtain material with progressive quality degradation, the seeds were moistened to 12% moisture content (wet basis) for 31 storage days. Seven kilograms of rapeseed (about 12 L) were stored in quasi-anaerobic conditions in 20 L glass containers. The tests were conducted at a temperature of approximately 21 °C and ca. 82% relative humidity. Rapeseed storage parameters were selected to obtain spoilage progression based on previous experience and data from the literature. Every 24 h, samples of volatile organic compounds for the chromatographic tests and e-nose analyses were collected from the headspace over the seed deposit with the use of a special system of silicone tubes and valves. Simultaneously, seed samples were collected for the analysis of ergosterol levels [14].

### 2.2. Ergosterol Analysis

Ergosterol was determined according to the procedure developed by Abramson and Smith [21] and Pronyk et al. [22]. A brief description of the method can be found in a previous study conducted by Gancarz et al. [6].

### 2.3. SPME/GC-MS

The GC-MS analyses were performed with the use of a Trace GC Ultra gas chromatograph (ThermoFisher Scientific, 81 Wyman St, Waltham, MA, USA) coupled with an ITQ 1100 mass spectrometer (ThermoFisher Scientific, 81 Wyman St, Waltham, MA, USA) according to the procedure described by Lippolis et al. [23]. Volatile compounds were collected from the headspace by solid phase micro-extraction (SPME) [24]. The chromatographic analyses were carried out using the SPME fiber 50/30 µm Divinylbenzene/Carboxen/Polydimethylsiloxane (DVB/CAR/PDMS), Stableflex (2 cm) 24 Ga (Sigma Aldrich, Poznan, Poland). The fiber with the adsorbent was placed in a measurement chamber (for 30 min) with a mixture of volatile organic compounds emitted by the stored rapeseed, channeled through a special valve. Passive SPME adsorption was used for sampling. Next, it was transferred into a GC injector for 5 min for desorption of volatile organic compounds. The injection port equipped with a 0.75 mm i.d. liner was maintained at 250 °C in the splitless mode. A Zebron ZB-5Msplus Capillary GC 30 m × 0.25 mm × 0.25 µm capillary column was used. The analyses were carried out at programmed temperature values: an initial temperature of 60 °C for 5 min, from 60 to 250 °C at 5 °C/min, from 250 to 270 °C at 10 °C/min, and the final temperature for 5 min. The helium flow rate was kept constant at 2.2 mL/min. The temperature of the transfer line and ion source was 280 °C. The electron impact ionization (EI+) mode with an electron energy value of 70 eV was applied. The mass spectrometer collected data in the full scan mode (scan ranges: 35–390). The procedure was also described by Rusinek et al. [25].

### 2.4. Electronic Nose

The Agrinose device, i.e., the electronic nose designed and constructed at the Institute of Agrophysics, Polish Academy of Sciences in Lublin [6], was used in the study. The device is based on a matrix of eight MOS (metal oxide semiconductor) sensors (TGS2600—general air contaminants, hydrogen and carbon monoxide; TGS2602—ammonia, hydrogen sulfide, high sensitivity to VOC and odorous gases; TGS2603—odors generated from spoiled foods; TGS2610—LP gas and butane; TGS2611—natural gas and methane; TGS2612—methane, propane and butane; TGS2620—solvent vapors, volatile vapors and alcohol; AS–MLV-P2—CO, butane, methane, ethanol and hydrogen). The Agrinose was previously used in analyses of bread spoilage, controlled baking processes, rancidity of edible oils and seed spoilage. Compared to previous studies, the device was modified by replacing sensor TGS2611 E00 (sensitivity to natural gas) with sensor TGS2603 for detection of food spoilage, which significantly improved the sensitivity of the device in terms of detection of VOCs in biological materials (see: Rusinek et al. [14]). The measurement cycle according to the sample protocol consisted of 10 s baseline purge, 60 s sample draw-in, and 140 s sample purge. Analog signals were converted to digital signals by means of DasyLab software. The sensorgrams obtained were converted to the ∗.xls format and analyzed using Statistica software (version 12.0, StatSoft Inc., Tulsa, OK, USA).

### 2.5. Three-Parameter Method for Generation of Smellprints

To date, the maximum sensor response parameter Δ*R/R_max_*, i.e., the maximum value of the change in resistance in the case of MOS or CP (conducting polymer) sensors, is most commonly used for generation of smellprints [25]. The authors developed a three-parameter method for generation of smellprints based on another two parameters, which was used for the first time on rapeseed [13]. These include the so-called response time *T_R_*, which is the time until achievement of the maximum response, and the cleaning time *T_CL_*, indicating the time of removal of molecules from the sensor’s active surface, i.e., the time from achievement of the maximum response Δ*R/R_max_* to half of its value. The parameters were measured on each storage day. Next, the results were statistically analyzed to identify a parameter that described the changes in the smell of the spoilage food most accurately.

### 2.6. Chemometrics

The analysis of variance, simple correlations and analysis of the main components were carried out at the significance level α = 0.05 using Statistica software (version 12.0, StatSoft Inc., Tulsa, OK, USA). The principal components analysis (PCA) analysis was performed to determine the relationship between the sensor response Δ*R/R_max_*, *T_R_*, and *T_CL_* for the eight sensors used in the study, the volatile compounds and ergosterol. The average values of parameters obtained from three replicates for each day of the experiment were used for PCA analysis. The optimal number of the principal components obtained in the analysis was determined based on the Cattel criterion. A data matrix with 32 rows (days of storage and control) and 44 columns (1 column—storage time, 24 columns—sensors responses, 18 columns—chemical compounds, 1 column—ergosterol) was constructed to determine the ability of the Agrinose to describe the qualitative degradation of the seeds. The input matrix was scaled automatically.

## 3. Results and Discussion

### 3.1. Ergosterol Content

**Figure 1:**
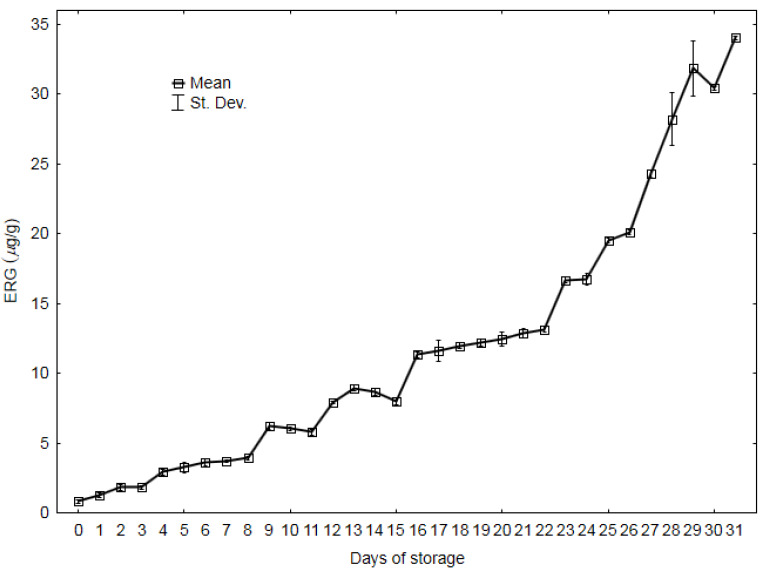
Ergosterol content (ERG) in rapeseed during 31 days of storage.

The ergosterol content was determined in triplicate. Figure 1 presents the averaged course of changes in ergosterol with standard deviations as a function of storage time, from the zero sample (control) to the sample from day 31, when the experiment was completed. The content of ergosterol increased over time during the experiment [6,26]. Before day 5, its content did not reach 3 μg/g, which is a threshold value for the suitability of materials for consumption [27].

Exceeding this value implies that the pressed oil can only be used for technical purposes, e.g., as biodiesel. Up until 22–23 days, the ergosterol value increased almost linearly, and this was followed by an intense increase [18]. Values over 30 μg/g were recorded on the last days of the experiment.

### 3.2. GC-MS Analysis

**Figure 2:**
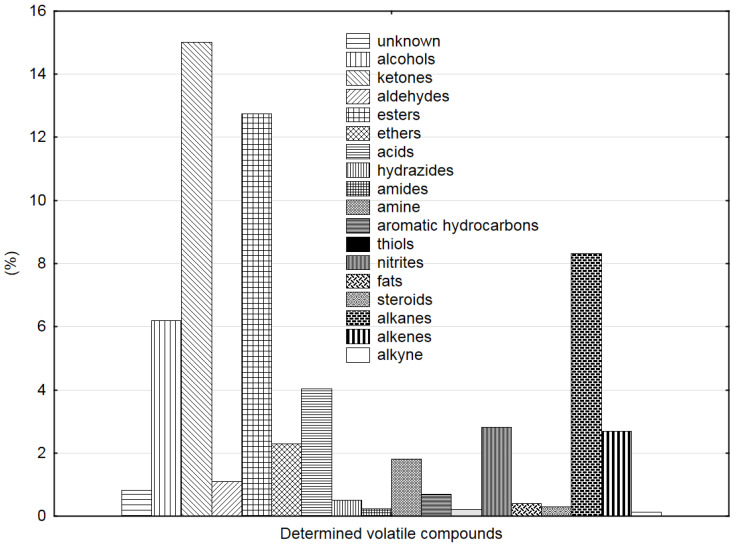
Percentage share of the main groups of volatile compounds in rapeseed during all days of storage.

The analysis of the chromatograms generated for the individual seed samples facilitated the identification of over 60% of the volatile organic compounds emitted during the experiment, and the assignment of them to major groups [28]. Figure 2 presents a cumulative graph of the percentage share of the chemical compounds detected during the 31-day rapeseed storage until complete degradation of the quality. The Wiley 138 library, with the highest quality of match, was used to identify the compounds [29]. The compounds were assigned to the main chemical groups of VOCs and presented in percentage charts. The detected compounds were grouped and their collective contents were shown in percentages for each storage day. Ketones, esters, alkanes, alcohols and acids accounted for the highest percentages during the experiment. A steady upward trend was noted for alcohols and alkenes (see Figure S1. Supplementary Materials); the increase in their content was positively correlated with seed degradation, reflected by the growing ergosterol level [30].

The upward trend in the level of alcohols and alkenes is most probably associated with the fermentation process accompanying seed quality degradation. The presence of this phenomenon was confirmed by an organoleptic analysis performed by Kubiak and Mikrut [31]. The seed deposit emitted an increasingly intense alcoholic odor with the progression of the degradation process.

### 3.3. Electronic Nose Analysis

**Figure 3:**
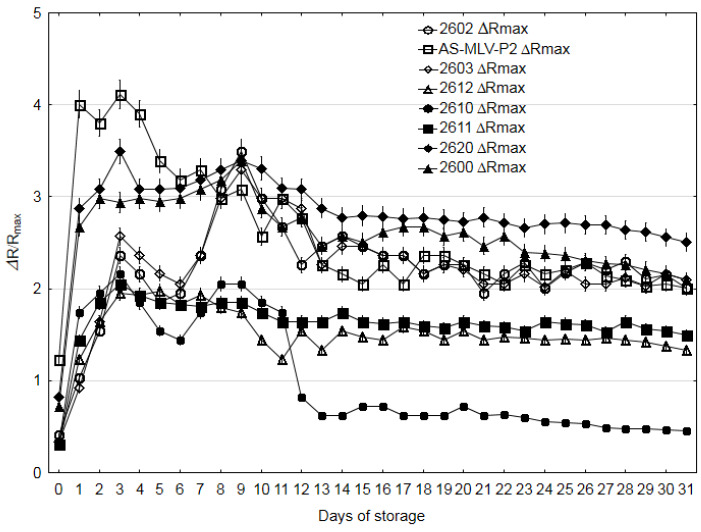
Maximum responses (Δ*R/R_max_*) of rapeseed during 31 days of storage.

**Figure 4:**
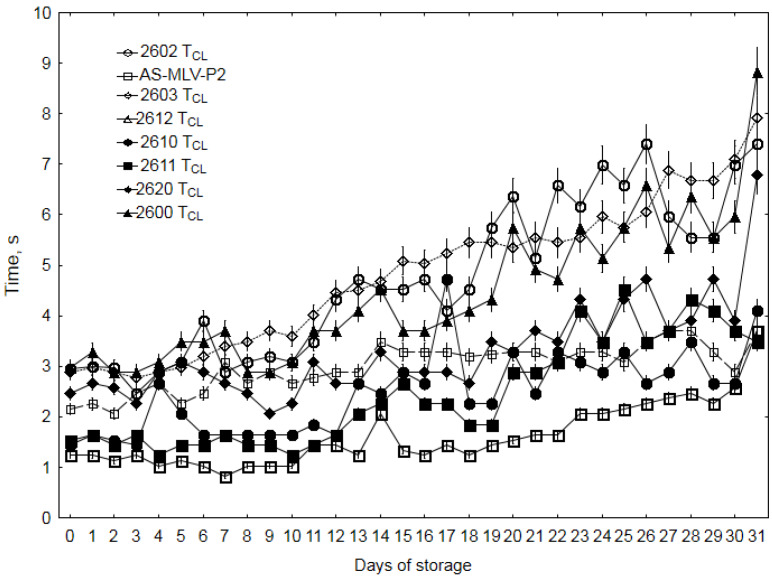
Cleaning time (*T_CL_*) of rapeseed during 31 days of storage.

The VOC analysis carried out using the electronic nose yielded the values of the response time *T_R_*, maximum response Δ*R/R_max_* and cleaning time *T_CL_* as a function of storage time (see Figure 3 and Figure 4, and Figures S2 and S3 in Supplementary Materials), as in the study conducted by Paolesse et al. [32]. The electronic nose response is stable and of a good magnitude (Figure S4 in Supplementary Materials). A clear trend can be noted in the case of the cleaning time *T_CL_* and the maximum response Δ*R/R_max_.* Initially, Δ*R/R_max_* increased to the value of 2–4 Δ*R/R_max_* for the individual sensors and then systematically declined from days 3–4 to the end of the experiment (Figure 3). This phenomenon was described by Gancarz et al. [6]. It was associated with a slow process of loss of fungal metabolic activity [33]. A reverse trend was evident in the case of the cleaning time *T_CL_*.

This parameter systematically increased for all sensors from the beginning of the experiment (Figure 4). The time of removal of molecules from the sensor’s active surface is associated with the intensity of the odor bouquet [13]. In the case of rapeseed spoilage, the intensity of the odor varied from the specific smell of good-quality seeds (ERG ≤ 3 μg/g) through musty to moldy odors (ERG > 3 μg/g). The initial seed-specific odor was hardly perceptible and pleasant for the observer. The musty and fermentative smell was very intense at a distance of several tens of centimeters away from the container aperture. The experienced researchers (in this case, the authors of the work) perceived this type of smell as unacceptable and suffocating [31,34]. Another trait besides the unacceptable nature of the odor was its high intensity. The large amount of molecules of the musty and fermentative smell that were adsorbed on the active surface of the sensor needed a long time to be removed after the measurement, resulting in a longer time of the resistance decline. This allows a conclusion that the greater number of molecules prolongs the time taken by the sensor to achieve the baseline characteristic of the atmosphere in which the sensor was calibrated [13].

In the same supply and signal amplification conditions, TGS2600 (sensitivity to general air contaminants), TGS2602 (high sensitivity to VOC and odorous gases) and TGS2603 (sensitivity to odors of spoiled foods) generate the highest signal values (Figure 4). This suggests that they are appropriate tools for the diagnostics of oilseed spoilage in terms of the Δ*R/R_max_* and *T_CL_* parameters. To answer the question whether the new three-parameter method for generation of smellprints was validated by the qualitative measurements of the oilseed degradation, the principal component analysis (PCA) was performed for the Δ*R/R_max_* and *T_CL_* parameters, which changed according to a specific algorithm as a function of storage time. In this case, the *T_R_* parameter, whose variability did not exhibit a specific trend, did not prove useful; therefore, it was not subjected to the PCA analysis (see Figure S2 in Supplementary Materials).

**Figure 5:**
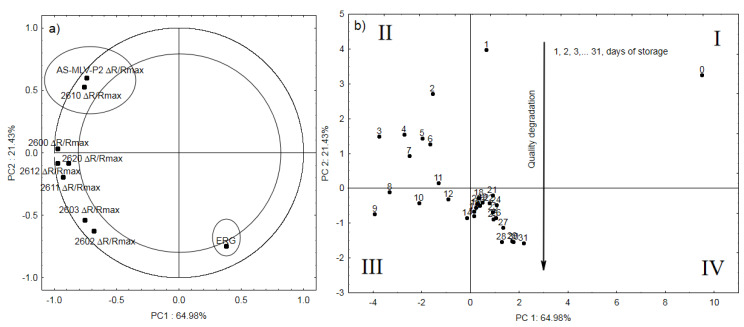
(**a**) location of loading vectors relative to the first two principal components (PC1, PC2) from eight sensor readings for Δ*R/R_max_* and ergosterol obtained for 31 days of storage; (**b**) plot of two principal component scores (PC1, PC2) from eight sensors for Δ*R/R_max_* readings and ergosterol obtained for 31 days of storage.

Figure 5a shows the relationships between the maximum sensor responses (Δ*R/R_max_*) and qualitative seed degradation reflected in the ergosterol content [6,35]. The negative PC1 values (64.98%) indicate the sensor response, while the positive values show changes in the ergosterol content occurring throughout the experiment. As demonstrated by the analysis, two of the eight sensors (marked with an ellipse) are negatively correlated with the changes in the ergosterol level in the case of Δ*R/R_max_*. One of the sensors is AS—MLV—P2, which is used for the measurement of volatile organic compounds. It is made using MEMS (Micro Electro Mechanical Systems) technology. This type of sensor is characterized by low power consumption as well as rapid response, and has sensors based on conducting polymers [25]. Figure 5b shows a projection of the cases on the PC1 and PC2 planes. As shown by the analysis, the quarters of the graph marked with Roman numerals sequentially describe the seed quality degradation: I—control sample and storage day 1; II—days 2–7; III—days 8–12; and IV—days 13–31. In general, it can be concluded that the PC2 component reflects the trend in the qualitative degradation expressed by the ergosterol level.

**Figure 6:**
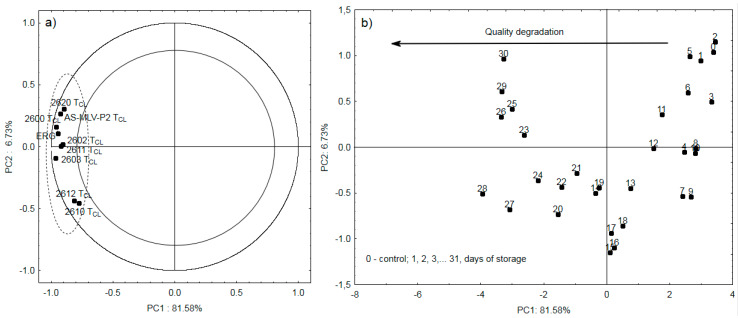
(**a**) location of loading vectors relative to the first two principal components (PC1, PC2) from eight sensor readings for *T_CL_* and ergosterol obtained for 31 days of storage; (**b**) plot of two principal component scores (PC1, PC2) from eight sensors for *T_CL_* readings and ergosterol obtained for 31 days of storage.

Figure 6a shows the correlation between the cleaning time *T_CL_* and the ergosterol level for the eight electronic nose sensors. As demonstrated in the Figure, there is a strong positive correlation between *T_CL_* and ergosterol. In contrast to the Δ*R/R_max_* parameter, *T_CL_* describes the qualitative seed degradation out of one hundred percent in the case of all eight sensors.

*T_CL_* seems to be the most appropriate of the three parameters proposed in the study for description of the quality changes in oilseeds. Nevertheless, the use of two or, in some cases, even three parameters determined during one measurement will ensure greater precision of the digital description of odors. Figure 6b presents the projection of cases on the PC1 and PC2 planes. The graph indicates that the first principal component PC1 describes 81.58% of the changes in the oilseeds stored for 31 days in the experiment. The positive PC1 values describe the slow increase in the ERG content of the first several days, whereas the negative values indicate a substantial increase in this marker until the end of the experiment (Figure 1). 

**Figure 7:**
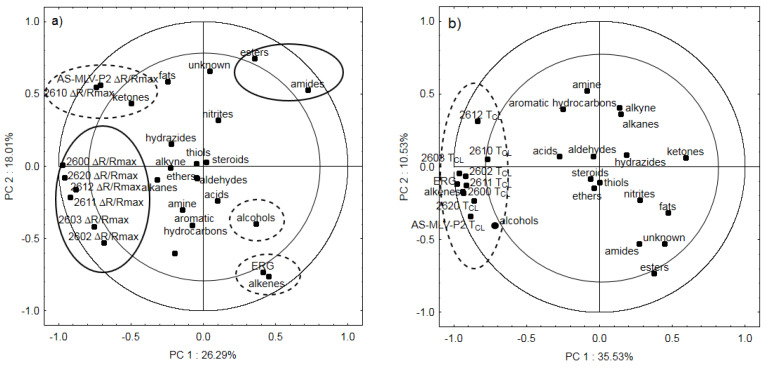
(**a**) location of loading vectors relative to the first two principal components (PC1, PC2) from eight sensor readings for Δ*R/R_max_*, ergosterol, and chemical groups of compounds obtained for 31 days of storage; (**b**) location of loading vectors relative to the first two principal components (PC1, PC2) from eight sensor readings for TCL, ergosterol, and chemical groups of compounds obtained for 31 days of storage.

The last analysis shows the projection of the variables on the PC1 and PC2 planes for the groups of chemical compounds, ergosterol, eight Δ*R/R_max_* parameters (Figure 7a), and eight *T_CL_* parameters (Figure 7b). In both cases, the negative values of the first principal component describe the response of the sensors to the changes in seed quality. It can be seen that parameter Δ*R/R_max_* (Figure 7a) is significantly negatively correlated with the content of ergosterol and alkenes, in the case of sensors AS—MLV—P2 and TGS2610, respectively. It is also more weakly correlated with alcohols (dashed-line ellipses in the Figure) and with esters and amides, in the case of sensors TGS2600, TGS2602, TGS2603, TGS2611 and TGS2620 (solid-line ellipses in the Figure).

Figure 7b shows a strong positive correlation between the cleaning time *T_CL_* and changes in the ergosterol content [22], as well as the presence of alcohols and alkenes. Figure 7a,b summarizes the investigations, and the correlations presented therein unambiguously validate the use of the additional parameter for the digital description of the odor of spoiled oilseeds [36].

Rapeseed (*Brassica napus* L.) is a popular oilseed plant in global oilseed production. Proper seed storage is key to the quality and shelf life of the oil pressed from this raw material. Oil pressed from seeds already in the first stage of deterioration, despite the absence of mold, was found to be unfit for consumption, despite the lack of color difference compared to oil pressed from seeds in good condition [15,18]. The content of ergosterol did not reach 3 μg/g before day 5, which is the threshold value for the suitability of materials for consumption [6,26,27]. However, not only oilseed rape is subject to storage. Edible oil is produced from other oilseeds, such as quince tree, safflower, fennel-flower, cuckoo-flower, tarweed, lallemantia, sea-buckthorn, borage, evening primrose, mustard, and others, which are subject to the storage process and may therefore deteriorate, similar to rapeseed. Oil obtained from these deteriorated seeds will not be fit for human consumption. Therefore, the results of the presented research for rapeseed can be helpful in the case of other oilseeds. They can also be useful in controlling the quality of stored seeds, and thus the quality of edible oil extracted from these seeds, which will reduce economic losses associated with degradation of oil quality [15,37].

## 4. Conclusions

The three-parameter method for generation of an electronic smellprint can be an alternative to the current widely used single-parameter methods. Two of the three parameters proposed describe changes in the degradation of oilseed quality accurately. The cleaning time was most strongly correlated with the markers of quality changes, i.e., the content of ergosterol and the presence of alcohols and alkenes as markers of the qualitative degradation of rapeseed. The three-parameters method can improve the accuracy of the digital description of odor intensity, and thus improve the precision of VOC measurements using an electronic nose without the need for interference in the device hardware. The test results confirm the opinion that the three-parameter method should be further tested on other biomaterials, to obtain optimal and universal indicators for assessment of their quality.

## Supplementary Materials

The following are available online at https://www.mdpi.com/1424-8220/20/11/3135/s1, Figure S1: Alcohols and alkenes as the function of days storage, Figure S2: Time reaction of sensors—*T_R_* as a function of time storage, Figure S3: Scheme of a typical sensorgram with three parameters for metal oxide semiconductor (MOS), Figure S4: Typical response graph for metal oxide semiconductor (MOS).

## References

[1] Marek G., Dobrzański B., Oniszczuk T., Combrzyński M., Ćwikła D., Rusinek R. (2020). Detection and Differentation of Volatile Compound Profiles in Roasted Coffee Arabica from Diffrent Countries Using an Electronic Nose and GC-MS. Sensors.

[2] Gębicki J., Szulczyński B. (2018). Discrimination of selected fungi species based on their odour profile using prototypes of electronic nose instruments. Measurement.

[3] Szczurek A., Maciejewska M., Bąk B., Wilde J., Siuda M. (2019). Semiconductor gas sensor as a detector of Varroa destructor infestation of honey bee colonies—Statistical evaluation. Comput. Electron. Agr..

[4] Garbacz M., Malec A., Duda-Saternus S., Suchorab Z., Guz Ł., Łagód G. (2020). Methods for Early Detection of Microbiological Infestation of Buildings Based on Gas Sensor Technologies. Chemosensors.

[5] Torri L., Piochi M. (2016). Sensory methods and electronic nose as innovative tools for the evaluation of the aroma transfer propierties of food plastic bags. Food Res. Int..

[6] Gancarz M., Wawrzyniak J., Gawrysiak-Witulska M., Wiącek D., Nawrocka A., Tadla M., Rusinek R. (2017). Application of electronic nose with MOS sensors to prediction of rapeseed quality. Measurement.

[7] Bonah E., Huang X.Y.R., Aheto J.H., Osae R., Golly M. (2019). Electronic nose classification and differentiation of bacterial foodborne pathogens based on support vector machine optimized with particle swarm optimization algorithm. J. Food Process. Eng..

[8] Liu H., Zhu W., Han Y., Yang Z., Huang Y. (2019). Single-Nanowire Fuse for Ionization Gas Detection. Sensors.

[9] Fan S., Li Z., Xia K., Hao D. (2019). Quantitative and Qualitative Analysis of Multicomponent Gas Using Sensor Array. Sensors.

[10] Kim J.-H., Mirzaei A., Kim H.W., Kim H.J., Quoc Vuong P., Kim S.S. (2019). Novel X-Ray Radiation Sensor Based on Networked SnO_2_ Nanowires. Appl. Sci..

[11] Jha S., Lathrop S., Nabrzyski J., Ramakrishnan L. (2019). Incorporating Scientific Workflows in Computing Research Processes. Comput. Sci. Eng..

[12] Sabilla S.I., Sarno R., Triyana K. (2019). Optimizing Threshold Using Pearson Correlation for Selecting Features of Electronic Nose Signals. Int. J. Intell. Syst..

[13] Gancarz M., Nawrocka A., Rusinek R. (2019). Identification of volatile organic compounds and their concentrations using a novel method analysis of MOS sensors signal. J. Food Sci..

[14] Rusinek R., Gancarz M., Krekora M., Nawrocka A. (2019). A novel method for generation of a fingerprint using electronic nose on the example of rapeseed spoilage. J. Food Sci..

[15] Rusinek R., Siger A., Gawrysiak-Witulska M., Rokosik E., Malaga-Toboła U., Gancarz M. (2019). Application of an electronic nose for determination of pre-pressing treatment of rapeseed based on the analysis of volatile compounds contained in pressed oil. Int. J. Food Sci. Tech..

[16] Jia Y., Xiuzhen G., Shukai D., Pengfei J., Lidan W., Chao P., Songlin Z. (2015). Electronic nose feature extraction methods: A Review. Sensors.

[17] Rusinek R., Kobyłka R. (2014). Experimental study and discrete element method modeling of temperature distributions in rapeseed stored in model bin. J. Stored Prod. Res..

[18] Gawrysiak-Witulska M., Siger A., Rudzińska M., Stuper-Szablewska K., Rusinek R. (2018). Effect of self-heating on the processing quality of rapeseed. Int. Agrophys..

[19] Muniz-Wypych A.S., Costa M.M., Oliveria A.R.S., New P.M., Schober S., Mittelbach M., Ramos L.P., César-Oliveira M.A.F. (2017). Phenolic compounds obtained from alkyl oleates as addi-tives to improve the oxidative stability of methyl rapeseed biodie-sel. Eur. J. Lipid Sci. Technol..

[20] Gugala M., Sikorska A., Findura F., Kapela K., Malaga-Tobola U., Zarzecka K., Domanski L. (2018). Effect of selected plant preparations containing biologically active compounds on winter rape (Brassica napus L.) yielding. Appl. Ecol. Env. Res..

[21] Abramson A., Smith D.M. (2003). Determination of ergosterol in canola (Brassica napus L.) by liquid chromatograph. J. Stored Prod. Res..

[22] Pronyk C., Abramson D., Muir W.E., White N.D.G. (2006). Correlation of total ergosterol levels in stored canola with fungal deterioration. J. Stored Prod. Res..

[23] Lippolis V., Pascale M., Cervellieri S., Damascelli A., Visconti A. (2014). Screening of deoxynivalenol contamination in durum wheat by MOS-based electronic nose and identification of the relevant pattern of volatile compounds. Food Control.

[24] Jeleń H.H. (2003). Use of solid phase microextraction (SPME) for profiling fungal volatile metabolites. Lett. Appl. Microbiol..

[25] Gancarz M., Wawrzyniak J., Gawrysiak-Witulska M., Wiącek D., Nawrocka A., Rusinek R. (2017). Electronic nose with polymer-composite sensors for monitoring fungal deterioration of stored rapeseed. Int Agrophys..

[26] Ng H.-E., Raj S.S.A., Wong S.H., Tey D., Tan H.-M. (2008). Estimation of fungal growth using the ergosterol assay: A rapid tool in assessing the microbiological status of grains and feeds. Lett. Appl. Microbiol..

[27] Gawrysiak-Witulska M., Siger A., Rusinek R. (2016). Degradation of tocopherols during rapeseed storage in simulated conditions of industrial silos. Int. Agrophys..

[28] García-González D.L., Aparicio R. (2010). Coupling MOS sensors and gas chromatography to interpret the sensor responsesto complex food aroma: Application to virgin olive oil. Food Chem..

[29] Rusinek R., Gancarz M., Nawrocka A. (2019). Application of an electronic nose with novel method for generation of smellprints for testing the suitability for consumption of wheat bread during 4-day storage. LWT-Food Sci. Technol..

[30] Magan N., Evans P. (2000). Volatiles as an indicator of fungal activity and differentiation between species, and the potential use of electronic nose technology for early detection of grain spoilage. J. Stored Prod. Res..

[31] Kubiak A., Mikrut Z. (2005). Rapeseed quality assessment using artificial nose and neural networks. Automation.

[32] Paolesse R., Alimelli A., Martinelli E., Di Natale C., Fanelli C. (2006). Detection of fungal contamination of cereal grain samples by an electronic nose. Sensor Actuat B- Chem..

[33] Weikl F., Ghirardo A., Schnitzler J.P., Pritsch P. (2016). Sesquiterpene emissions from Alternaria alternate and Fusarium oxysporum: Effects of age, nutrient availability, and co-cultivation. Sci. Rep..

[34] Kubiak A., Wenzl T., Ulberth F. (2012). Evaluation of the quality of postharvest rapeseed by means of an electronic nose. J. Sci. Food Agric..

[35] Delgado-Rodriguez M., Ruiz-Montoya M., Giraldez I., Lopez R., Madejon E., Diaz M.J. (2012). Use of electronic nose and GC-MS in detection and monitoring some VOC. Atmos. Environ..

[36] Kubiak A. (2016). Rapeseed Analysis by an Electronic Nose. Electronic Noses and Tongues in Food Science.

[37] Rusinek R., Rybczyński R., Gawrysiak-Witulska M., Nogala-Kałucka M., Siger A., Tys J. (2012). The process parameters for non-typical seeds during simulated cold deep oil expression. Czech J. Food Sci..

