# Supplementary material for: Influence of Changes in the Level of Volatile Compounds Emitted during Rapeseed Quality Degradation on the Reaction of MOS Type Sensor-Array
Source: Sensors (Basel). 2020 Jun 1;20(11):3135. doi: 10.3390/s20113135 (PMC7309047; doi:10.3390/s20113135)
Supplement: Supplementary file 1 [file sensors-20-03135-s001.pdf]

sensors

Supplementary Materials

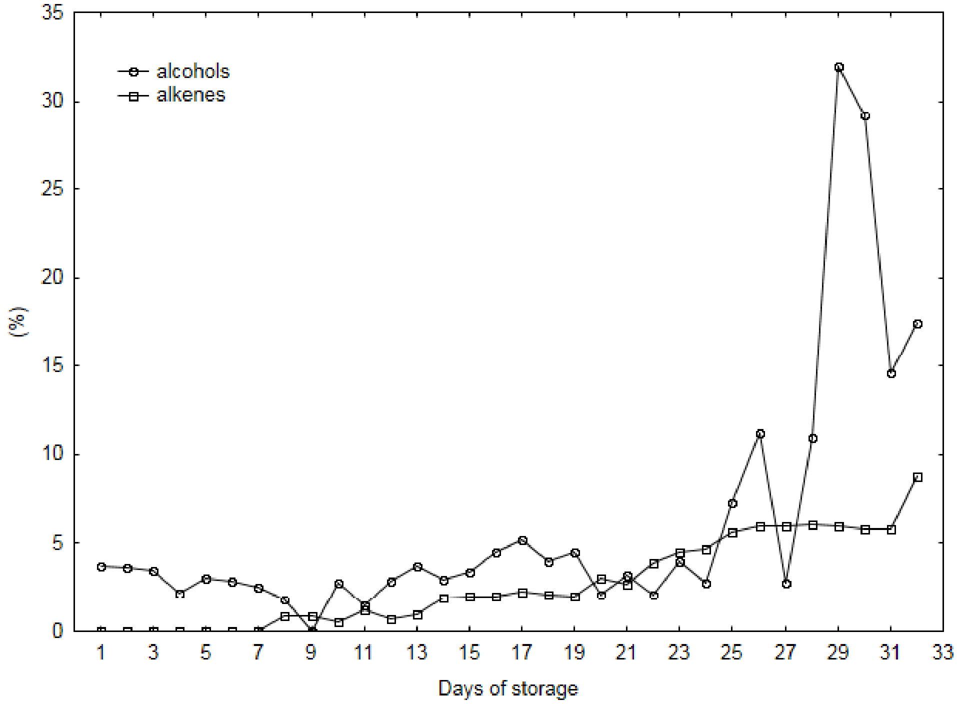

Figure S1. An alcohols and alkenes as the function of days storage.

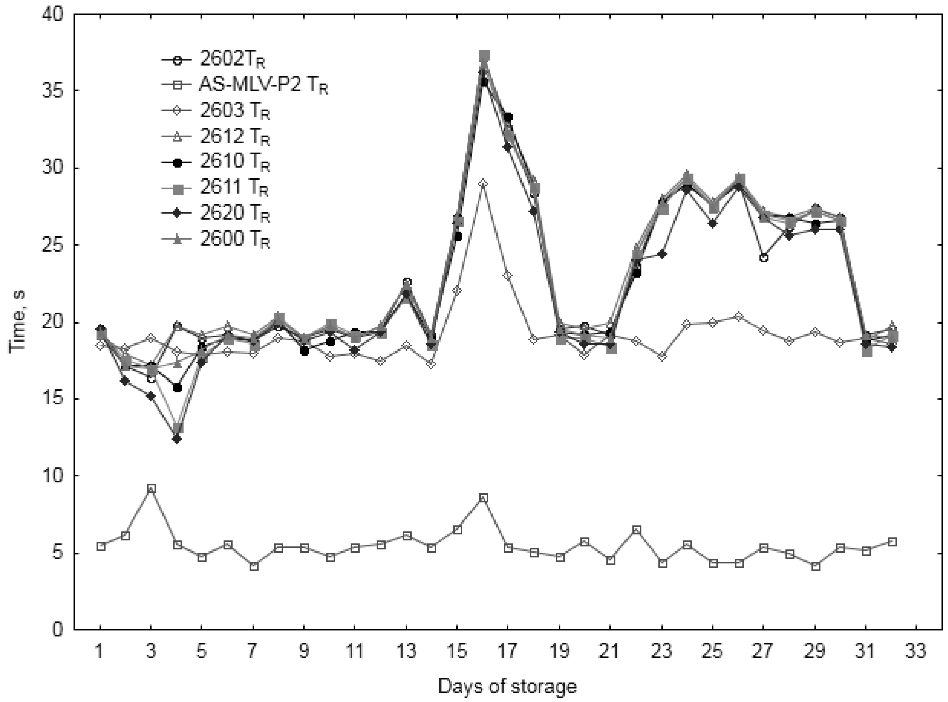

Figure S2. Time reaction of sensors - T_R as a function of time storage.

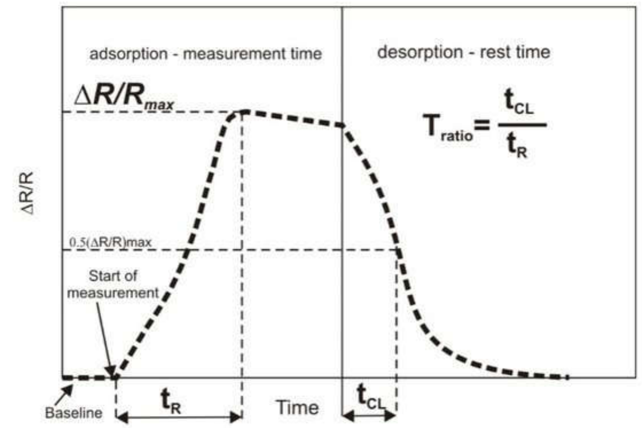

Figure S3. Scheme of a typical sensorgram with three-parameters for metal oxide semiconductor (MOS).

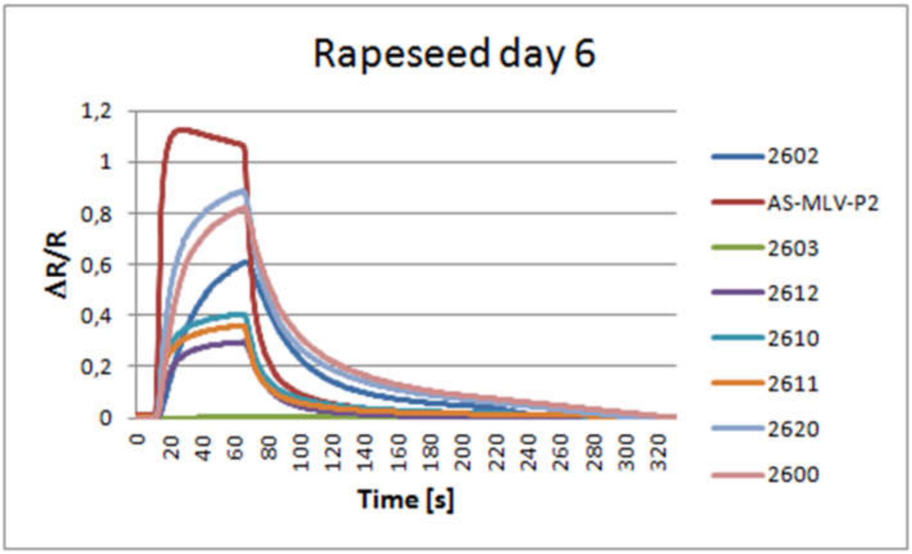

Figure S4. Typical response graph for metal oxide semiconductor (MOS).
